# The Royal Institute of Public Health

**Published:** 1920-12-04

**Authors:** 


### THE ROYAL INSTITUTE OF PUBLIC HEALTH.

The twenty-sixth annual congress of the Institute, which will be of an international character, will be held in 1921, on the invitation of the University, in the city of Geneva, from Tuesday, May 10, to Monday, May 16, inclusive. Further particulars can be obtained from the Secretary, 37 Russell Square, W.C. 1.

